# A Role for Hedgehog Signaling in the Differentiation of the Insertion Site of the Patellar Tendon in the Mouse

**DOI:** 10.1371/journal.pone.0065411

**Published:** 2013-06-10

**Authors:** Chia-Feng Liu, Andrew Breidenbach, Lindsey Aschbacher-Smith, David Butler, Christopher Wylie

**Affiliations:** 1 Division of Developmental Biology, Cincinnati Children’s Hospital Research Foundation, Cincinnati, Ohio, United States of America; 2 Biomedical Engineering Program, School of Energy, Environment, Biological and Medical Engineering, University of Cincinnati, Cincinnati, Ohio, United States of America; Indiana University School of Medicine, United States of America

## Abstract

Tendons are typically composed of two histologically different regions: the midsubstance and insertion site. We previously showed that Gli1, a downstream effector of the hedgehog (Hh) signaling pathway, is expressed only in the insertion site of the mouse patellar tendon during its differentiation. To test for a functional role of Hh signaling, we targeted *the Smoothened (Smo*) gene *in vivo* using a Cre/Lox system. Constitutive activation of the Hh pathway in the mid-substance caused molecular markers of the insertion site, e.g. type II collagen, to be ectopically expressed or up-regulated in the midsubstance. This was confirmed using a novel organ culture method *in vitro*. Conversely, when *Smo* was excised in the scleraxis-positive cell population, the development of the fibrocartilaginous insertion site was affected. Whole transcriptome analysis revealed that the expression of genes involved in chondrogenesis and mineralization was down-regulated in the insertion site, and expression of insertion site markers was decreased. Biomechanical testing of murine adult patellar tendon, which developed in the absence of Hh signaling, showed impairment of tendon structural properties (lower linear stiffness and greater displacement) and material properties (greater strain), although the linear modulus of the mutant group was not significantly lower than controls. These studies provide new insights into the role of Hh signaling during tendon development.

## Introduction

Tendons play an essential role in the musculoskeletal system. They transmit force from muscles to bones during body movements and bear huge tensile forces during exercise. As such, tendons are easily injured but difficult to repair. Their healing can be slow and complete recovery is rare [1]. Thus, it is necessary to develop more efficient treatments for tendon injuries. However, the development of new therapies has been impeded due to limitations in our understanding of tendon development.

Insertion sites are the points at which tendons and ligaments attach to bone or cartilage, and are the regions of maximum stress concentration, which makes them prone to injury [2]. Two primary types of insertion sites are present in mammals: fibrocartilaginous (direct) and fibrous (indirect). A recent study showed that the formation of fibrous insertions is regulated by Parathyroid hormone-related protein (PTHrP) [3]. However, the mechanisms regulating the differentiation and formation of fibrocartilaginous insertion sites are still not clear.

In a previous study, we screened for active cell signaling pathways during development of the mouse patellar tendon. We showed that cells in the insertion site of the patellar tendon respond to Hedgehog (Hh) signaling during late fetal and early postnatal life, a period when cell proliferation is ending and tenocyte cell differentiation is beginning [4]. Cells in the midsubstance of the tendon were negative for the Hh reporter signal, suggesting that Hh signaling may be required for some aspects of insertion site differentiation.

Hh signaling is a key cell-signaling pathway that plays many essential roles in both embryonic and post-embryonic development [5]. Two Hh ligands, sonic hedgehog (Shh) and Indian hedgehog (Ihh), have been shown to regulate the development of musculoskeletal system [6]. Both of these ligands bind to the membrane-bound receptor Patched1 (PTCH1) to elicit their functions. Binding of the Hh ligand to PTCH1 reverses the inhibition of the transmembrane protein, smoothened (SMO), which then activates a cascade of signals in the responding cells. SMO-mediated signal transduction regulates the activities of GLI-Kruppel family member (GLI) transcription factors. Therefore the activation of *Gli* expression, in particular *Gli1*, serves as a marker of active Hh signaling [7], [8].

The specific expression of *Gli1* in the insertion site suggests the hypothesis that Hh signaling pathway is involved in the differentiation of the fibrocartilaginous insertion site. To test this hypothesis, we used both *in vivo* and *in vitro* approaches to study the function of Hh signaling in the differentiation of the fibrocartilaginous insertion site of the mouse patellar tendon, with the goal of understanding which aspects of fibrocartilaginous insertion site differentiation are regulated by Hh signaling during the normal development. We show that the active ligand is Indian hedgehog, and present both *in vitro* and *in vivo* data that Ihh signaling is required for normal differentiation of the fibrocartilaginous insertion site.

## Results

### Activation of Hedgehog Signaling Pathway in the Scx-positive Cells Altered the Expression Pattern of Insertion Site Markers

**Figure 1:**
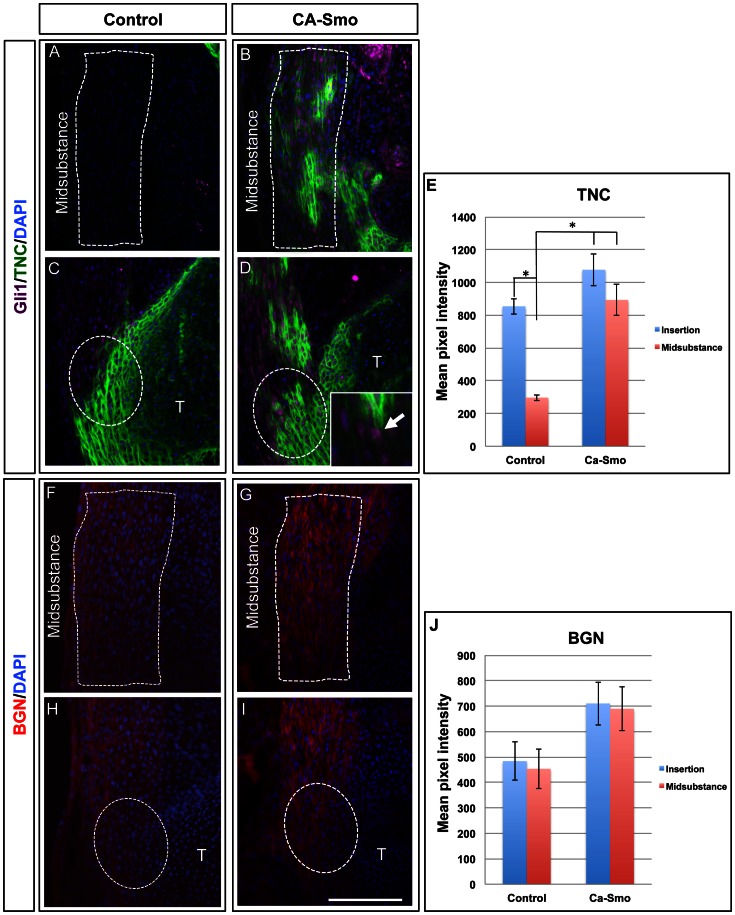
Expression of insertion site markers in the control and constitutively active Smo (CA-Smo) mice at E17.5. Panels A–D show double immunohistochemistry of GLI1 (purple in nucleolus) and TNC (green) in control (A and C) and CA-Smo (B and D) sagittal sections of knee joints. Panel F–I show the immunohistochemistry of BGN in control (F and H) and CA-Smo (G and I) sagittal sections of knee joints. Panel E and J show semi-quantitation of TNC and BGN staining by measurement of pixel intensity. The expression of GLI1 and TNC was ectopically present in the midsubstance of CA-Smo mice whereas the expression of BGN was up-regulated in the midsubstance of CA-Smo mice at E17.5. The Inset in D shows a higher magnification image of GLI1 staining in the nucleolus (purple, arrowed). White dotted lines outline the midsubstance in A,B, F and G, and the insertion site in C,D, H and I. Error bars represent standard errors of the mean and the asterisk represents statistical significance (*p*<0.05). Blue staining shows nuclear staining using DPAI. T = Tibia. Scale bar = 100 µm.

To understand the role of Hh signaling pathway in the development of the patellar tendon, we targeted Smoothened (Smo), a key receptor of Hh signaling pathway on the cell membrane. We first carried out gain-of-function assays *in vivo* by generating mice in which all tenocytes expressing Scleraxis (Scx) also expressed a constitutively active mutant of Smo (see materials and methods for details). In the insertion sites of control patellar tendons, the downstream Hh target *GLI1* was expressed only in the insertion sites (Fig. 1A and C), but extended into the mid-substance of the constitutively active Smo (CA-Smo) patellar tendons (Fig. 1B and D, purple staining in the nuclus and insect in 1D). This confirmed the activation of Hh signaling throughout the tendon.

**Figure 2:**
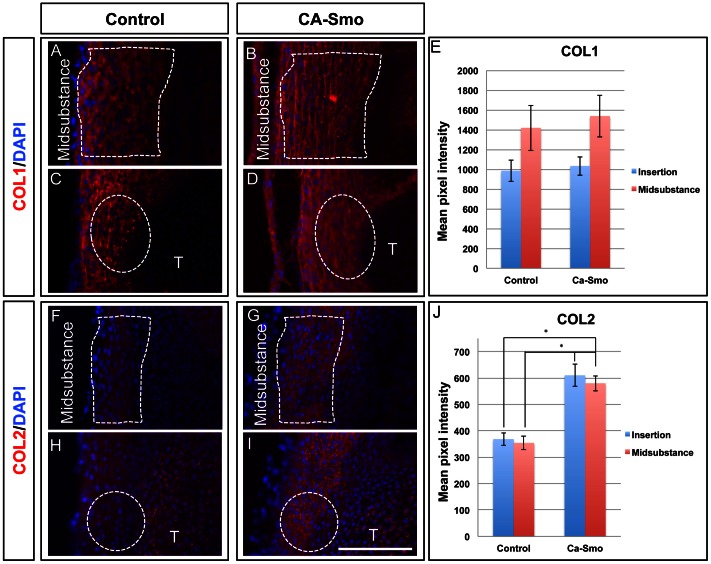
Expression of collagens in control and constitutively active Smo (CA-Smo) patellar tendons at E17.5. Panels A–D show immunohistochemistry of COL1 in control (A and C) and CA-Smo (B and D) sagittal sections of knee joints. Panels F–I show immunohistochemistry of COL2 in control (F and H) and CA-Smo (G and I). Activation of Smo in the tendon does not affect the expression of COL1 but causes ectopic expression of COL2 in the midsubstance. Panel E and J show pixel intensity measurements of COL1 and COL2 staining. COL2 was up-regulated in the CA-Smo mice throughout whole tendon. White dotted line marks the region of midsubstance. White dotted circle marks the insertion site. Error bar represents standard error and the asterisk represents statistical significance (*p*<0.05). Blue staining shows nuclear staining using DAPI. T = Tibia. Scale bar = 100 µm.

Tenascin-C (TNC) and biglycan (BGN) are two extracellular matrix components whose expression is normally concentrated at the insertion site of the patellar tendon, and whose first appearance there coincides with Hh signaling at the insertion site [4]. To test whether Hh signaling activates these insertion site markers, we stained saggittal sections from control and CA-Smo patellar tendons with antibodies specific for TNC and BGN. In embryonic day 17.5 (E17.5) animals, TNC, was expressed at higher levels in the mid-substance of the CA-Smo patellar tendons than in control tendons (Fig. 1A,B and E). Expression of the insertion site marker BGN also increased from low levels in the midsubstance and insertion site of the control animal to higher levels in both the midsubstance and insertion site of the CA-Smo animals (Fig. 1F–I). We also carried out semi-quantitative analysis of pixel intensities in the fluoresence images using the Zeiss Axiovision software. The data showed that the expression of TNC was significantly increased in the midsubstance of CA-Smo animal compared to that in the control animals (Fig. 1E, *p*<0.05). We also observed an increased in expression of BGN in the midsubstance of CA-Smo (mean = 690) tendons compared to controls (mean = 454), although the difference was not statistically significant (Fig. 1J, *p* = 0.08). COL1, normally expressed throughout the tendon, was unaffected (Fig. 2A–D). However, expression of type 2 Collagen (COL2), a later stage marker of the insertion site, showed dramatically up-regulated expression throughout the tendon of the CA-Smo mice (Fig. 2F–I). Pixel intensity measurements confirmed these observations (Figs. 2E and J respectively). These data show that activation of Hh signaling throughout the patellar tendon activates genes normally expressed in the insertion site, to which Hh signaling is normally restricted.

### IHH, but not SHH, is Present in the Differentiating Insertion Site of the Patellar Tendon

**Figure 3:**
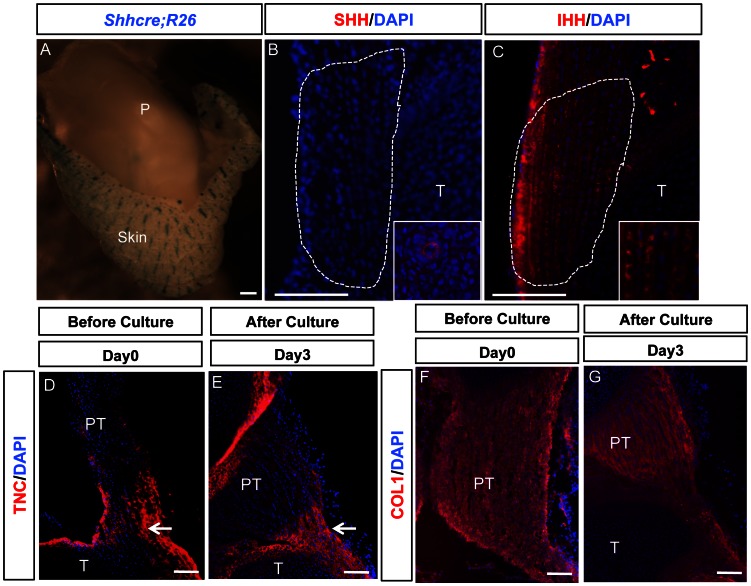
IHH protein expression in the tibial insertion of the patellar tendon *in vivo*, and expression of tenocyte and insertion site markers in patellar-tendon-tibia (PTT) organ culture. (A) beta-gal staining (blue) of ShhCre; R26 knee joints. (B) Immunohistochemistry to show absence of SHH protein in P1 patellar tendon insertion site. Inset shows positive staining of SHH in the epithelium of a blood vessel in the same section as (B). (C) Immunohistochemistry of IHH protein in P1 patellar tendon insertion site. Inset shows higher magnification of IHH staining. (D–E) Tenascin-C staining before (D) and after (E) culture of P3 tendons. (F–G) Collagen I staining before (F) and after (G) culture. Expression of both COL1 and TNC was maintained in PTT organ culture. See text for details. P = Patella. PT = Patellar tendon. T = Tibia. Arrow = insertion site. Scale bar = 100 µm.

Although we previously showed that Hh signaling is active in the insertion site [4], no studies have identified the ligand involved. To assay for the presence of Shh, we used a reporter mouse line generated by crossing mice transgenic for Shh-Cre to ROSA-26 mice, and stained for beta-galactosidase activity using X-gal. No activity was seen in the tendon insertion site (Fig. 3A). This result was confirmed by the absence of immunostaining at postnatal day 1 (P1) using an antibody against SHH (Fig. 3B). However, IHH expression in the insertion site was confirmed by immunohistochemistry on sagittal sections from P1 patellar tendons using an antibody which was specific for IHH (Fig. 3C). The pattern of IHH staining was very similar to that previously reported for Gli1 [4] and shown in the present study (Fig. 1A and C). These data show that IHH is the active Hh ligand present in the insertion site.

CA-Smo animals die at birth due to respiratory failure. In order to study the role of IHH at postnatal stages, we generated a rapid functional assay for signaling pathways in the developing patellar tendon using an organ culture system previously used in this laboratory for both embryo slice cultures [9], [10], [11], [12] and developing intervertebral discs [13] to test whether or not IHH can regulate the differentiation of insertion site.

Fig. 3D–G shows sections of tendons before and after culture, stained with an antibody against tenascin-C (TNC), which is expressed *in vivo* at higher levels at the insertion site and collagen type 1 (COL1), which is expressed throughout the tendon [4]. These data show that the overall pattern of tendon differentiation was maintained in culture for these periods.

**Figure 4:**
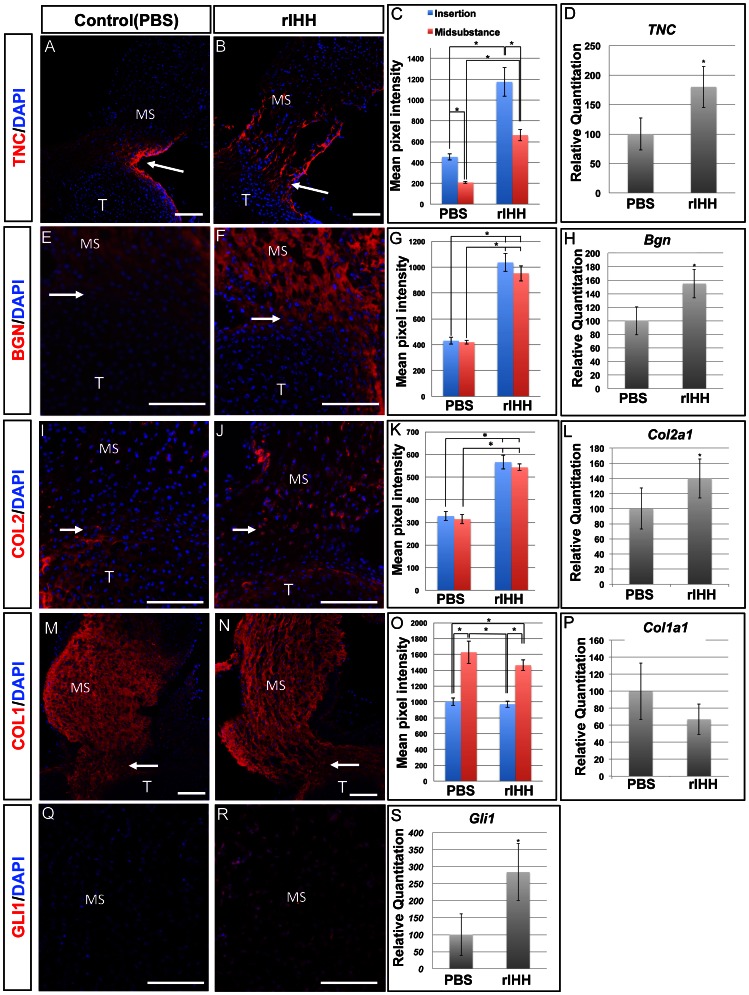
Expression of tenocyte and insertion site markers after administration of 500 ng/ml rIHH in PTT organ culture. Panel A–B show immunohistochemistry of TNC on PTT culture in PBS control (A) and rIHH (B). Panel E–F show immunohistochemistry of BGN in PTT culture in PBS control (E) and rIHH (F). Panel I–J show immunohistochemistry of COL2 in PTT culture in PBS control (I) and rIHH (J). Panel M–N show immunohistochemistry of COL1 in PTT culture in PBS control (M) and rIHH (N). Panel Q–R show immunohistochemistry of GLI1 in PTT culture in PBS control (Q) and rIHH (R). Panels C, G, K and O show pixel intensity measurements of TNC(C), BGN(G), COL2(K) and COL1(Q) staining. Panels D, H, L, P and S show expression levels of *Tnc* (D), *Bgn* (H), *Col2a1*(L), *Col1a1* (P), *Gli1* (S) mRNAs by Q-PCR. The expression of TNC, BGN, COL2 and GLI1 was up-regulated in tendons after rIHH treatment in culture, but there was no significant difference in COL1 expression between the control and rIHH treatment groups. See text for details. MS = midsubstance. T = Tibia. Arrow = insertion site. Scale bar = 100 µm. Error bar represents standard error and the asterisk represents statistical significance (*p*<0.05).

After tendon differentiation was confirmed in cultures, protein and mRNA levels of genes enriched in the insertion site were examined by immunohistochemistry and Q-PCR. Fluorescence images were subjected to semi-quantitative analysis using the Zeiss Axiovision software for pixel intensity measurements. After treating cultures of P3 patellar tendons for three days with recombinant IHH (rIHH, 500 ng/ml), expression levels of TNC, BGN, and COL2 were up-regulated and extended into the mid-substance. (Fig. 4A–C, 4E–G, and 4I–K, respectively). Quantitation of the staining showed that the expression of TNC, BGN and COL2 were all increased after treating with rIHH for three days (Fig. 4C, G and K, *p*<0.05). TNC had higher levels of expression in the insertion sites of both control and rIHH treated groups (Fig. 4C, *p*<0.05). While COL2 and BGN both showed higher expression levels in the rIHH treatment group, there was no obvious difference between midsubstance and insertion site in either the control or rIHH treated groups (Fig. 4G and K). mRNA levels of *Tnc*, *Bgn* and *Col2* were all up-regulated after treating P3 patellar tendon cultures with rIHH (Fig. 4D, H, and L). COL1, the tendon marker normally expressed throughout the tendon was unaffected by IHH treatment (Fig. 4M–P). GLI1, a known downstream target of IHH, was ectopically expressed in the midsubstance in the rIHH treated group (Fig. 4Q–R). The mRNA levels of *Gli1*, were also elevated when assayed by Q-PCR (Fig. 4S). These data showed that IHH can actively regulate differentiation of the insertion site in the growing patellar tendon.

### Absence of Hedgehog Signaling Pathway in the Scx-positive Cells Affected Differentiation of the Fibrocartilaginous Insertion Site *in vivo*

**Figure 5:**
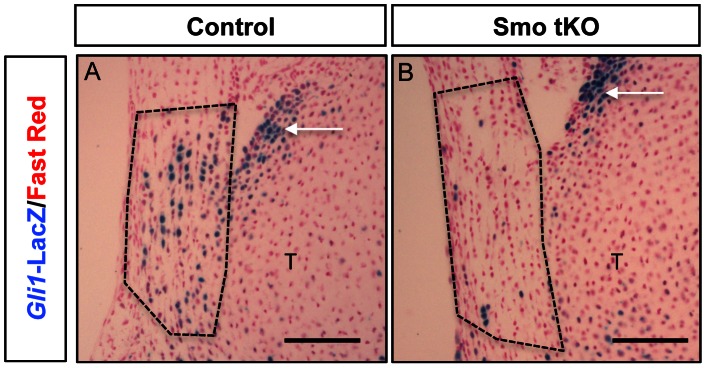
Hedgehog signaling (Hh) is down-regulated at the insertion in *ScxCre;Smo^f/−^(*Smo tKO). (A) Control mice (B) Smo tKO. The reduction of beta-gal staining (blue) in the Smo tKO indicated that Hh signaling was decreased in the insertion site. See text for details. Cell nuclei are stained using fast red. Dotted line circle = insertion site. Arrow = articular cartilage. Scale bar = 100 µm.

We next carried out loss-of-function experiments *in vivo* by excising *Smo* in tenocytes using the ScxCre mouse line. This was done in a Gli1-beta-galactosidase (*Gli1-LacZ*) reporter mouse line, so that the effect on Hh signaling could be assayed directly in the experimental tendons by X-gal staining. In control animals, X-gal staining was seen mainly in the tendon-to-bone insertion site and articular cartilage of the tibia (Fig. 5A). However in the Smo tissue-specific KO animal (*ScxCre;Smo^f/−^* or Smo tKO), X-gal staining was restricted to a few cells in the insertion site but still present in the articular cartilage of the tibia (arrowed in Fig. 5B). In addition, the expression of *Smo* and *Gli1* mRNA was undetectable in the Smo mutation by Q-PCR (Data not shown). Thus, Cre-mediated targeting of Smo in the tenocytes caused reduced Hh signaling in the tendon-to-bone insertion site.

**Figure 6:**
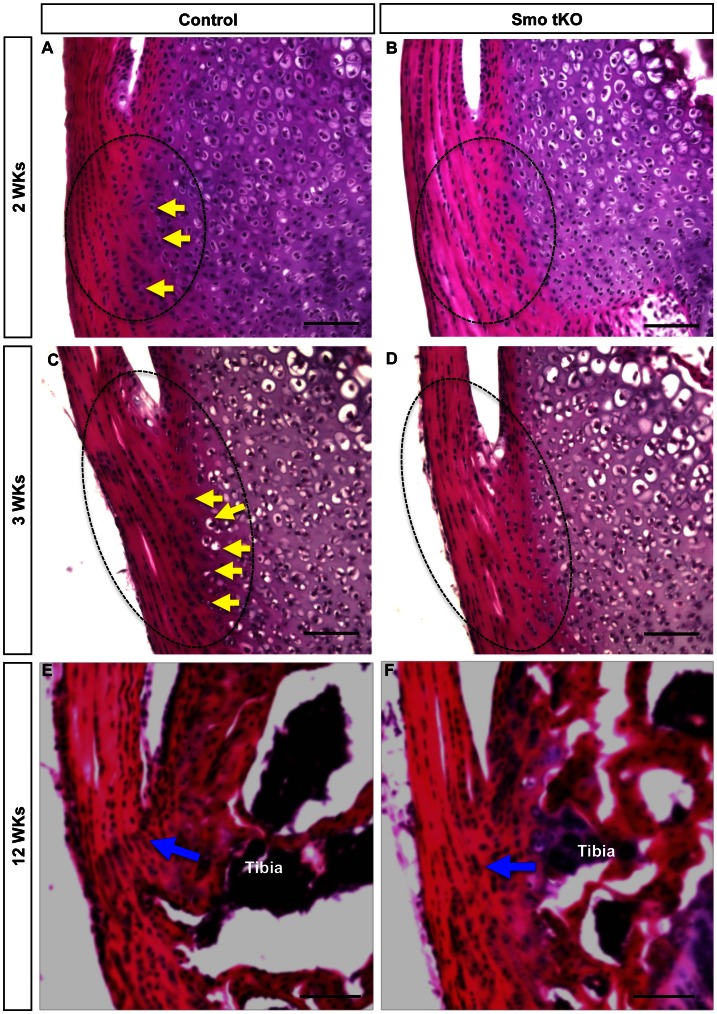
H&E staining of the tibial insertion site of patellar tendon of control and *ScxCre;Smo^f/−^(*Smo tKO). Panel A–B show H&E staining of the sagittal sections of 2 weeks (2 wks) old control (A) and Smo tKO (B). Panel C–D show H&E staining of the sagittal sections of 3 wks old control (C) and Smo tKO (D). Panel E–F show H&E staining of the sagittal sections of 12 wks old control (E) and SmotKO (F). There are fewer chondrogenic cells (yellow arrow) in the tibial insertion site of patellar tendons in Smo tKO animals, and the tidemark of the Smo tKO mice is absent. See text for details. Yellow arrow = advanced differentiated cells. Blue arrow = tidemark. Scale bar = 100 µm.

**Figure 7:**
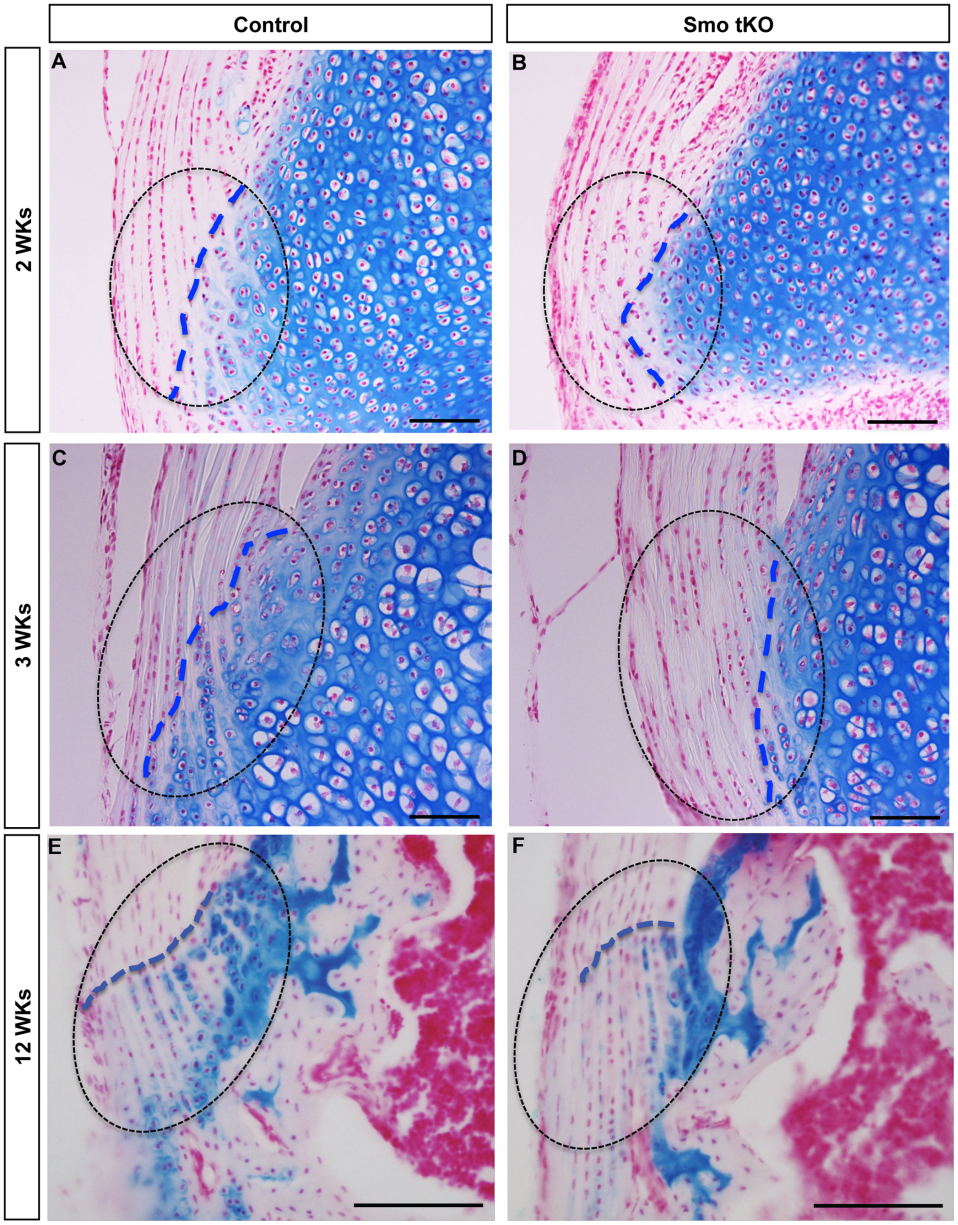
Alcian blue staining is decreased in the tibial insertion sites of patellar tendons of *ScxCre;Smo^f/−^(*Smo tKO). Shows alcian blue staining in insertion sites of control (A, C, and E) and Smo tKO (B, D, and F) patellar tendons at 2 wks (A and B), 3 wks (C and D) and 12 wks (E and F). See text for details. Dotted line outlines the insertion site. Blue dotted line shows the boundary between the tendon and tibial cartilage. See text for details. Cell nuclei are stained using fast red. Scale bar = 100 µm.

Control and Smo tKO tendons also showed changing histological differences with age. No dramatic early histologic differences were observed in H&E stained sections (P1–P7, data not shown). However, subtle changes could be seen by 2–3 weeks with more differentiated cells with larger rounded shapes in the control animals than in the Smo tKO (Fig. 6A–B, yellow arrows). At 3 weeks (3 wks), more differentiated cells within cartilaginous lacunae could be identified in the insertion sites of control animals. No such differentiated cells were observed in the Smo KO animal, suggesting that loss of Hh signaling caused reduced chondrogenesis of the insertion site (Fig. 6C–D, yellow arrows). This was supported by a reduction of highly sulfated cartilage GAGs in the mutant insertion sites, as seen by reduced Alcian blue staining (Fig. 7). In control insertion sites, the boundary between the tendon and tibial cartilage was broken up by tendon fibers extending into the tibial cartilage and chondrogenic tenocytes (Fig. 7A,C&E, blue dotted lines); however, this area of cell mixing was considerably reduced in the Smo tKO animals (Fig. 7B,D&F, blue dotted lines).

More obvious differences became visible with increasing age. At 12 weeks old the tidemark, which marks the boundary between the un-calcified and calcified fibrocartilage layers, can been seen in the insertion sites of the control animals but not in the Smo tKO mice (Figure 6E–F,blue arrow). These data suggest that the differentiation of fibrocartilage in the insertion site is controlled by the Hh signaling pathway.

**Figure 8:**
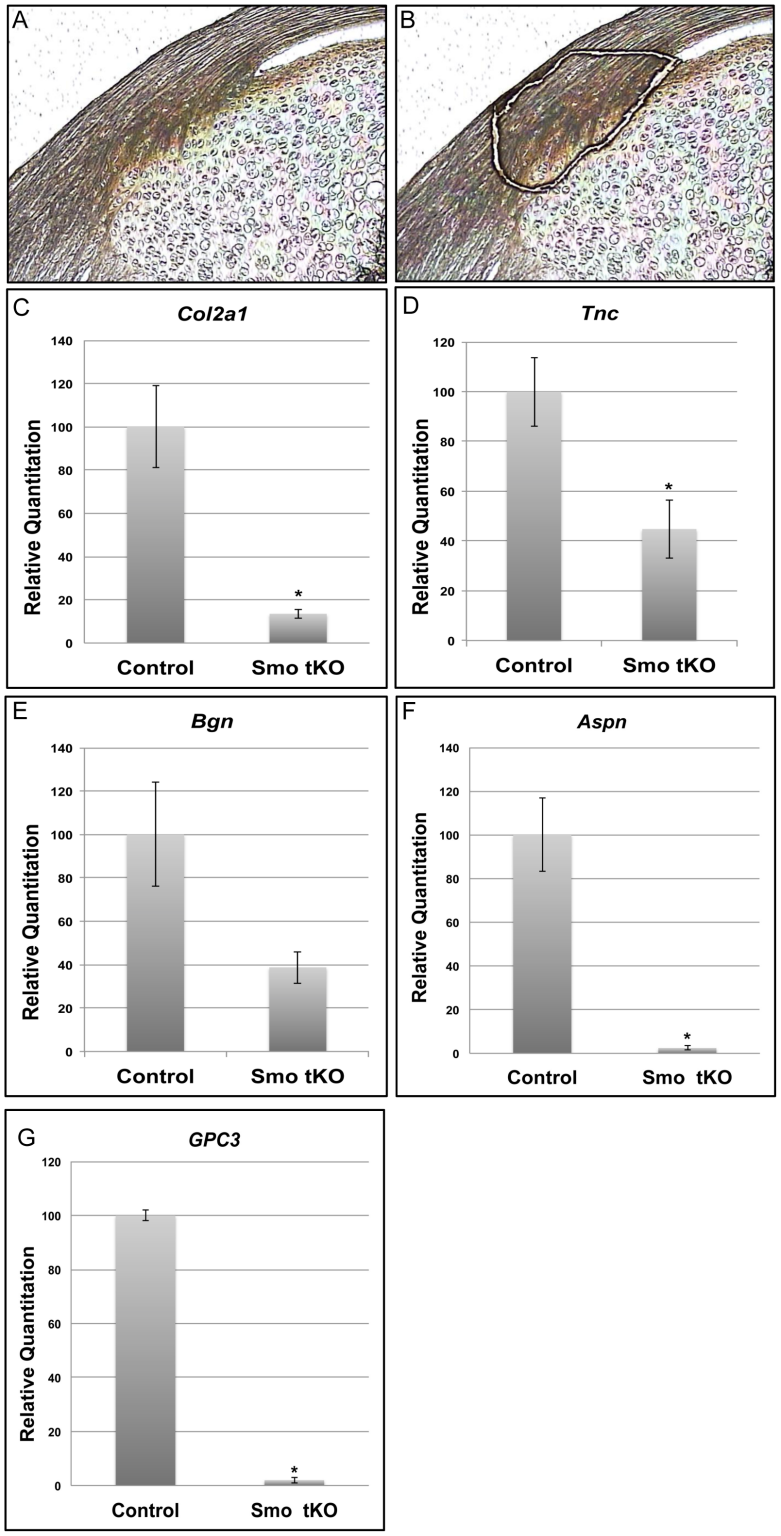
Expression of insertion site markers in the tibial insertion site of patellar tendon of *ScxCre;Smo^f/−^(*Smo tKO). Panels A–B show a mid-sagittal section of 2 wks old mouse patellar tendon before (A) and after (B) LCM dissection of the insertion site. Panels C–G show Q-PCR results of *Col2a1*(C), *Tnc* (D), *Bgn* (E) *Aspn* (F) and *Gpc3* (G). All these markers were reduced in the Smo tKO animals. See text for details. The asterisk represents statistical significance (*p*<0.05).

To understand what mechanisms required Hh signaling in the insertion, we used laser capture microscopy (LCM) to dissect insertion sites for RNAseq from cryostat sections of P14 patellar tendons of control (*Smo^f/+^* and *ScxCre;Smo^f/+^* ) and Smo mutants (*ScxCre;Smo^f/−^*)(Fig. 8A–B). P14 was used because it is the time when the morphology of a tibal insertion site can first be easily seen under a dissecting microscope. Data analysis of RNAseq results revealed that genes involved in the regulation of extracelluar matrix proteins were 1.5 fold down-regulated in the absence of Hh signaling pathway. These genes included *Col2a1*, a well-known insertion site marker (Table 1). This was confirmed using Q-PCR analysis (Fig. 8C, *p*<0.05). In addition, lumican (*Lum*), a small leucine-rich progeoglycan that associates with fibrillar collagens and regulates collagen fibrillogenesis, was also down-regulated in the Smo mutant (Table 1). Two other insertion markers, *Tnc* and *Bgn*, showed opposite effects. While *Tnc* expression decreased in the Smo tKO insertion, *Bgn* showed no such effect (Table 1). Q-PCR analysis confirmed these RNAseq results with a significant decrease in *Tnc* expression (Fig. 8D, *p*<0.05), but no significant difference in *Bgn* expression between control and Smo tKO tendons (Fig. 8E, *p* = 0.15). GO analysis of the RNAseq data revealed that genes involved in the mineralization process, glypcian 3 (*Gpc3*) and asporin (*Aspn*), were 2.5 times and 3.9 times decreased, respectively, in the absence of Hh signaling (Table 1). We comfirmed these findings with Q-PCR analysis (Fig. 8F&G). These data showed that mineralization of the insertion site was affected in the Smo tKO mice and may explain why the tidemark was less obvious in the Smo tKO sections (Fig. 6F).

**Table 1:** List of analyzed genes, RPKM value, fold difference and brief summary of function.

Gene name	Control RPKM	Smo tKO RPKM	Fold Change(Smo tKO/Control)	*p*-value	Function	
Asporin	16.19	6.63	−3.91	<0.001	leucine-rich repeat (LRR)	
Biglycan	1147.60	1371.00	1.20	<0.001	Small leucine-rich repeat proteoglycan (SLRP)	
Collagen, type II, alpha 1	1494.34	862.30	−1.70	<0.001	Collagen II protein	
*Gli1*	14.50	1.25	−12.46		Hedgehog downstream target	
Glypican 3	9.66	3.90	−2.46	<0.001	Glypican-related integral membrane proteoglycan family (GRIPS)	
Lumican	28.20	17.48	−1.75	<0.001	SLRP	
Runx2	22.60	12.79	−1.70	<0.001	Transcription factor	
Smoothened	36.49	0.73	−99.31	<0.001	Hedgehog signaling receptor	
Tenasin-C	9.77	8.53	−1.3	<0.001	Extracellular matrix (ECM) protein	

RPKM, reads per kilobase exon per million mapped sequences

### Absence of Hedgehog Signaling in Scx-positive Cells Affected Several Biomechanical Properties of the Mouse Patellar Tendon

**Figure 9:**
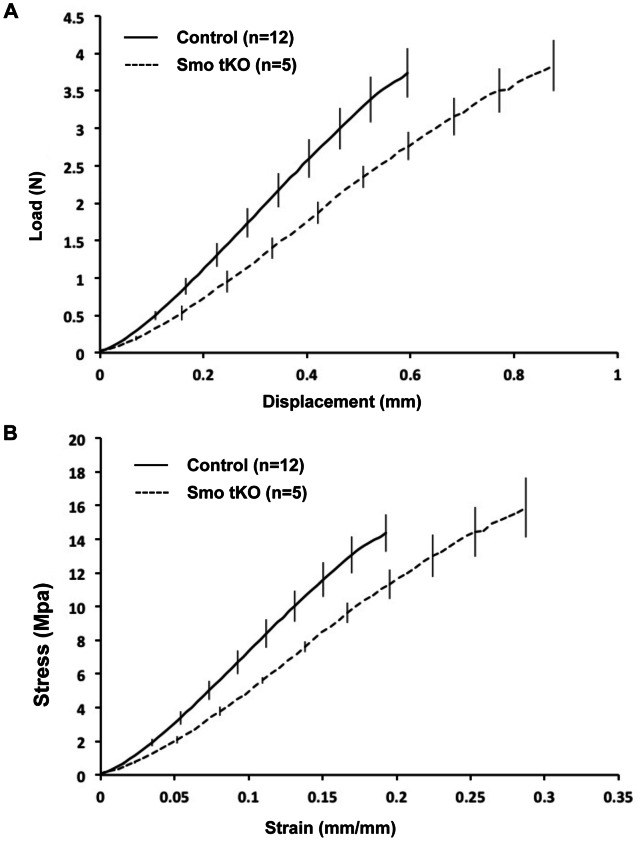
Mechanical failure curves of patellar tendons of the control and *ScxCre;Smo^f/−^(*Smo tKO) at 12 wks old. Adult Smo tKO patellar tendons displayed impaired structural (A) and material (B) properties compared to control animals. Smo tKO tendons showed decreased linear stiffness, increased displacement and increased strain (*p*<0.05). Linear modulus trended towards a decrease in Smo tKO animals; however, results were not statistically significant.

To determine if the function of the tendon-to-bone insertion site was affected by loss of Hh signaling, we carried out biomechanical testing in adult, 12-week old control (n = 12) and Smo tKO animal (n = 5). The mean body weight of control animals was greater than that of the Smo tKO animals, however control and Smo tKO tendons showed no differences in length (*p* = 0.89), width (*p* = 0.36) or thickness (*p* = 0.80) (Table 2). Furthermore, linear regression analysis did not reveal significant correlation between weight and any biomechanical or dimensional parameter, thus, allowing for fair comparison of tendon mechanical properties. Biomechanical testing showed that the Smo tKO patellar tendons displayed significantly lower linear stiffness (*p*<0.01) and significantly greater maximum displacement (*p*<0.01) compared to controls (Fig. 9A and Table 2). In addition, Smo tKO tendons also had significantly higher maximum strains than that in the control (*p*<0.01) (Fig. 9B and Table 2). Maximum failure force and stress were not significantly different between control and Smo tKO animals (Fig. 9A & B and Table 2). The mechanism of failure was localized to the insertion in 75% of the control animals and 60% of Smo tKO animals. However, logistic regression analysis showed no significant effect of the genotype on tendon failure mechanism. The biomechanics analysis demonstrated that the tensile properties of tendon were impaired in the absence of Hh signaling pathway, suggesting that the Hh pathway regulates the differentiation of the fibrocartilaginous insertion site throughout development, growth and maturation.

**Table 2:** Patellar tendon dimensions and biomechanical results of Smo tKO compared to control mice.

	Control (n = 12)	Smo tKO (n = 5)
**Linear Stiffness (N/mm)** a	7.97±0.23	6.23±0.22
**Maximum Failure Force (N)**	3.74±0.33	3.84±0.34
**Maximum Displacement (mm)** a	0.59±0.05	0.88±0.07
**Elastic Modulus (MPa)**	98.23±6.92	80.52±9.18
**Maximum Stress (Mpa)**	14.37±1.11	15.86±1.77
**Maximum Strain (mm/mm)** a	0.19±0.02	0.29±0.02
**Body Weight (g)**	27.28±0.90	23.34±1.12
**Length (mm)**	3.11±0.06	3.08±0.12
**Full Width (mm)**	1.07±0.05	1.04±0.06
**Dissected Width (mm)**	0.61±0.03	0.56±0.02
**Thickness (mm)**	0.43±0.02	0.44±0.03

a Significantly different between control and mutant (mean±SE, *p*<0.05).

## Discussion

Insertion site injuries are common and difficult to repair. An increased understanding of the molecular mechanisms of insertion site differentiation would provide useful information for future translational studies. Here we used mouse genetics and organ culture studies to study the role of Hh signaling during the differentiation of the fibrocartilaginous insertion site. We demonstrated that IHH was responsible for the activation of the Hh pathway in the tibial fibrocartilaginous insertion site. Activation of Hh signaling in whole tendon disrupted normal tendon development and caused up-regulated or ectopic appearance of insertion site markers in the midsubstance. Ablation of Hh signaling resulted in decreased expression of insertion site markers and affected chondrogenesis of the fibrocartilaginous insertion. Our data indicates that the Hh signaling pathway is required for normal differentiation of the patellar tendon fibrocartilaginous insertion site and may have implications in the development of other tendon-to-bone insertion sites. This study could also be important in developing strategies to improve treatment of insertional tendon injuries.

The maturation of insertion sites requires contributions from both tenocytes and chondrocytes [14], [15], as both of these cell populations produce and deposit essential extra cellular matrix constituents to form a proper attachment between tendon and bone. When we activated Hh pathway *in vivo* in the tenocyte population, the insertion site marker COL2 was ectopically expressed and TNC and BGN were up-regulated in the midsubstance of tendon at embryonic stage. In addition, in *vitro* organ culture treated with rIHH increased the expression of these insertion site makers in the mid-substance. These results demonstrate that Hh signaling controls the expression of insertion site components during tendon development. Studies have shown that TNC, BGN and COL2 are also associated with the development of cartilage [16], [17]. Therefore, Hh signaling in the tenocyte may function to regulate chondrogenesis in tenocytes in the insertion site.

Hh signaling may not be the only signal controlling expression of TNC and BGN, however. In tendons cultured in the absence of rIHH, mRNAs for *Bgn* and *Tnc* can still be detected (Fig. 4D–H). Although, Q-PCR analysis showed that both *Bgn* and *Tnc* mRNA were reduced in the Smo-targeted patellar insertion site *in vivo, Bgn* mRNA did not show statistically different between control and Smo tKO. Moreover, both *Tnc* and *Bgn* mRNA were not absent in Smo tKO. In addition, BGN and TNC proteins can still be seen by immunohistochemistry (data not shown). There are several possible explanations. First, Smo was only targeted in the tenocytes of the patellar tendon. Adjacent tissues, such as the tibial cartilage or connective tissue layers of the tendon itself, may also express these genes, since they are not uniquely expressed in tenocytes. Second, Gli1-positive cells were still seen, albeit in much reduced numbers, in the insertion sites of Smo-targeted patellar tendons (Fig. 5B). These could be due to incomplete *Smo* excision in tenocytes. Third, studies using rat and human tissue models have shown that Bgn and Tnc are also present in the midsubstance of tendon at adulthood [18], [19], [20]. Therefore other signaling pathways besides IHH could also control expression of these markers. Without more data we cannot distinguish among these possibilities. What is clear from the data is that genes known to be involved in fibrillogenesis, chondrogenesis and mineralization (*Runx2*, *Col2a1* and *Gpc3,* for example) were down-regulated in the insertion sites from Smo tKO animals. Thus, it is clear that the Hh signaling pathway participates in regulating these cellular events at the insertion site. Moreover, loss of Hh signaling eliminates the characteristic tidemark in H&E stained sections of the insertion sites.

Tendon biomechanical function relies on the proper composition and organization of extracellular matrix proteins, such as fibrillar collagens, e.g. COL1 and COL2, and proteoglycans, e.g. BGN. The evidence presented here shows decreased gene expression of insertion site-specific markers during early post-natal development of the Smo tKO animal. In addition, there were clearly disruptions in the organization of the insertion site relative to the formation of the tidemark (Fig. 6E–F) and localization of cartilage-specific proteoglycans (Fig. 7) that persisted into adult stages of maturation.

Biomechanical analysis demonstrated significantly lower linear stiffness in the Smo tKO tendon. This reduction was due to an increase in total tissue displacement rather than a decrease in failure load. As the normal tendon insertion is known to experience greater strain during failure than the tendon midsubstance [21], alterations in the organization of the tendon insertion site suggest that the increased strains in the mutants could have been localized to the altered insertion site. However, a limitation of this study is that we did not specifically measure localized strain patterns and cannot therefore ensure that loss of Hh signaling does not also result in non-local (e.g. midsubstance) biomechanical abnormalities. Nevertheless, this alteration in tissue mechanics is likely due to lower production of type II collagen and other essential proteoglycans associated with the absence of the Hh signaling pathway. It will be important to further investigate how the Hh signaling pathway regulates collagen fibrillogenesis at the tendon insertion during postnatal stages.

We found that IHH protein was present in the insertion site in a pattern similar to that of Gli1-LacZ [4]. This result indicates that IHH is the ligand responsible for regulating the expression of Gli1 in the insertion site. IHH signaling not only regulates chondrocyte proliferation in long bone development along with parathyroid-hormone-related protein (PTHrP) in the growth plate but also triggers the formation of the intramembranous bone collar near the diaphysis [22], [23], [24]. Since the growth plate at the end of each long bone is essential for bone lengthening, it is possible that IHH from the growth plate or from the articular cartilage acts on tenocytes in the insertion site during the formation of the fibrocartilage insertion site. Although it has been shown that targeted deletion of IHH caused short-limb dwarfism with decreased chondrocyte number and extensive hypertrophy [23], [25], to our knowledge, there has been no description of the effects on tendon or ligament insertions in the *Ihh* mutant.

In the clinic surgical repair of injured tendons often fails because a functional insertion site is never regenerated. Interestingly, the new insertion site after surgical reattachment is initially a fibrous insertion, since there is no cartilage present [26]. We observed that Hh signaling was decreased at the insertion site after P7, suggesting Hh signaling may not be present at the insertion in adulthood [4]. Hh signaling is known to be associated with age-related joint diseases [27], [28], [29]. A recent study also showed that delivery of an Ihh gene can induce chondrogenesis in human mesenchymal stem cells [30]. Thus, our results and others suggest that it may be necessary to supply Hh signaling after surgical repair to regenerate a functional insertion, and IHH may serve as an antagonist of aging and its associated diseases. However, we must overcome the challenge of delivering Hh signaling locally at the insertion site without affecting the midsubstance after surgical repair. It is also necessary to learn how different regions of tendons respond to signals during normal development as well as during the healing process [31]. It is worth noting here that the source of IHH during differentiation of the insertion site, the tibial cartilage which has ossified by the adult stage, would no longer be a source of Hh signals during tendon repair or grafting. Furthermore, the tenocytes that are chondrifed in response to Hh signaling will be replaced by bone via the actions of osteoclast and osteoblasts [32]. Therefore, simply supplying Hh signaling after surgical repair of injured tendon may not be sufficient to generate a new insertion site. Further studies investigating the functions of the Hh signaling pathway in the insertion site maturation and evaluating the application of Hh signaling to improve the healing process of injured tendon will enhance our understanding of how to regenerate rather than simply repair tendon after injury.

## Materials and Methods

### Ethics Statement

All procedures described were reviewed and approved by the Institutional Animal Care and Use Committee at CCHMC and were performed in accordance with the Guiding Principles for the Care and Use of Laboratory Animals.

### Animals, Breeding Scheme, and Mating

Scleraxis-Cre (ScxCre) mice were generously provided by Dr. Ronen Schweitzer and will be described elsewhere by his group [33]. *GT(ROSA)26Sor^tm1(smo/EYFP)Amc^*/J (SmoM2) mice, *Smo^tm2Amc^*/J (*Smo^f/f^*) and *Gli1^tm2Alj^/J* (Gli1-LacZ ) were obtained from Jackson Laboratory (SmoM2, stock number: 005130; Gli1-LacZ, stock number: 008211).

Mice expressing a constitutively active form of the Smoothened (Smo) protein in tenocytes were generated by crossing ScxCre with a dominant active allele of Smo mouse (SmoM2) (Figure S1) [34], [35]. This consitutively active expression of Smo is blocked by a loxP-flanked stop coden. ScxCre mice express Cre recombinase specifically in tendon and ligament cells and will remove the DNA sequence between the two loxPs [33], [36]. This constitutively activates the intracellular Hh signaling pathway in Scx-positive tenocytes (*ScxCre;SmoM2* or CA-Smo). SmoM2 littermates were used as controls to compare with CA-Smo mutants.

To generate Smo loss-of-function mice (*ScxCre;Smo^f/−^* or SmotKO), we crossed *ScxCre;Smo^+/−^* mice to *Smo^f/f^* mice following the standard breeding scheme (Figure S1) [37]. *Smo^f/+^*, *Smo^f/−^*, or *ScxCre;Smo^f/+^* littermates were used as controls to compare with Smo tKO. Females were housed with males and plug-checked next morning. Detection of a vaginal plug was considered as embryonic day 0.5. The joints of animals were collected at desired time points. CD1 mice were obtained from Charles River (strain code 022) to generate postnatal day 3 (P3) mice for organ culture. Genotyping was determined by PCR. Genotyping primers used for specific genes were as described: 5′-GAGTGAACGAACCTGGTCGAAATCAGTGCG-3′ and 5′-GCATTACCGGTCGATGCAACGAGTGATGAG-3′ for Cre genotyping; 5′-CCA CTGCGAGCCTTTGCGCTAC-3′ and 5′ CCCATCACCTCCGCGTCG CA-3′ for Smo wildtype allele; 5′- CTTGGGTGGAGAGGCTATTC-3′ and 5′ AGGTGAGAT GACAGGAGATC-3′ for Smo floxed allele; and 5′-GGGATCTGTGCCTGAAACTG-3′, 5′- TCTGCCAGTTTGAGGGGACGAC-3′ and 5′-AGGTGAGACGACTGCCAAGT-3′ for Gli1-LacZ genotyping. All experiments were performed on at least three to five animals for each control group and experimental group for each time point. Please refer to the specific experimental procedures and animal numbers below for detail.

### *In vitro* Organ Culture

Patellar tendons, together with patellas and proximal tibiae (PTT) were dissected from P3 CD1 mice. The isolated PTTs were put onto millicell® culture inserts (Millipore, Cat# PICM02150) pre-coated with collagen I (Millipore). Inserts were then placed into a 24-well plate with culture medium (Knockout™ DMEM,Gibco; 100 U/ml penicillin/Streptomycin, Gibco; 1× insulin-transferrin-sodium selenite supplement, Roche Diagnostics). Cultures were maintained in a 37°C, 5% CO_2_ incubator. For gain-of-function experiments, cultures were treated with 500 ng/ml recombinant Indian hedgehog (rIHH)(R&D, Cat# 1705-025/CF) or vehicle (PBS) for 72 hours. Medium was changed every 24 hours. Cultured tissues were collected and processed for analysis by histology, immunohistochemistry and Q-PCR. A total of twenty-one P3 CD1 mice were used for cultures. Experiments were performed in three biological experiments (n = 3).

### Histological Analysis

Tissues from both genders of embryos as well as postnatal day mice up to 2 weeks (2 wks) old were freshly embedded in OCT (Tissue-Tek) before snap freezing in liquid nitrogen. Tissues from both genders of mice 3 wks postnatally or older were fixed overnight in 4% paraformaldehyde at 4°C and then washed with PBS the next day. Washed knee joints were then treated with decalcification buffer (0.5M EDTA, pH 8.0 or 100 mM Tris pH 7.5, 10% EDTA-4 Na, and 7.5% polyvinyl pyrolidione (PVP)) at 4°C. After decalcification, samples were infiltrated with sucrose and embedded in OCT for sectioning. Samples were collected at E17.5 for Smo gain-of-function studies. Samples were collected at 2 wks, 3 wks and 12 wks for Smo loss-of-function studies. At least five animals were used for each experiment.

Embedded tissues were sectioned at 10 µm then mounted on slides. Slides from unfixed tissues were fixed with 4% paraformaldehyde, washed in PBS and then stained using Hematoxlin and Eosin (H&E). Slides from fixed tissues were washed in PBS and stained using H&E. Slides were then dehydrated and embedded in mounting medium Xylene (Fisher Scientific).

### Immunohistochemistry

Samples were isolated and embedded in OCT before snap freezing in liquid nitrogen. Ten (10 µm) cryostat sections were fixed with 2%–4% paraformaldehyde (PFA) for 10 minutes or 2% Trichloroacetic acid (TCA) for 6 minutes, washed in PBS, and permeabilized with PBST (0.1% Triton X-100 in PBS). Slides were then incubated in blocking solution (10% donkey serum or 10% donkey serum and 1% gelatin, 0.1% Triton X-100, and 4% BSA in PBS) for 1 h at room temperature. Sections were incubated with primary antibodies (see below) at 4°C for overnight. The next day sections were washed three times for 10 min each with PBST and PBS followed by incubation in the corresponding secondary antibodies. Sections were next washed three times with PBS for 10 minutes each and embedded in mounting medium with DAPI nuclear counterstain (VECTASHIELD®). Samples were collected at E17.5 for Smo gain-of-function studies. Samples were collected at 2 wks, 3 wks and 12 wks for Smo loss-of-function studies. At least five animals were used for each experiment.

The dilutions and sources of primary antibodies used were anti-Indian Hedgehog (1∶50, Abcam,Cat No. ab39634), anti-Snoic Hedgehog (1∶100, Abcam Cat No. ab50515), anti-collagen I (1∶200, Novus Biological Research, Cat No. NBP1-30054), anti-collagen II (1∶200, Novus Biological Research, Cat No. NB100-91715), anti-biglycan (1∶100, AbCam, Cat No. ab58562), anti-tenascin C (1∶1000, AbCam, Cat No. ab6346), and anti-GIL1 (1∶50,R&D, Cat No. AF3455). All secondary antibodies were purchased from Jackson ImmunoResearch Laboratories, Inc. and used according to manufacturer’s instructions.

### Semi-quantitative Analysis of Immunohistochemistry

Images were acquired at 20X using the same exposure settings for all samples within each experimental group. The mean intensity of fluorescence was measured using the outline tools and measurement in the AxioVision Rel 4.8.2.0 software (Carl Zeiss MicroImaging LLC, NY, USA) [4]. A total of 9 sections from 3 animals or 3 PTT cultures (n = 3) were analyzed per immunohistochemistry within each experiment group.

### Beta-Gal (X-Gal) Staining

For whole-mount 5-bromo-4-chloro-indolyl-β-D-galactopyranoside (X-gal, Invitrogen Cat No. B-1690) staining, knee joints were briefly fixed in 4% paraformaldehyde at room temperature and then washed three times in X-gal washing buffer for 5 minutes each (2 mM MgCl_2_, 0.02% NP-40 in PBS). Samples were then incubated in X-gal reaction substrate containing 2 mM MgCl_2_, 0.02% NP-40, 3.5 mM K ferrocyanide and 3.5 mM K ferricyanide overnight at 25°C in the dark. Next day, samples were washed with X-Gal washing buffer, post-fixed in 4% paraformaldehyde, washed with PBS, and preserved in PBS at 4°C. For section X-Gal staining, sections were fixed with 2% paraformaldehyde for 2 minutes at 4°C, washed in PBS, X-Gal washing buffer (2 mM MgCl_2_, 0.02% NP-40, 0.05% sodium deoxycholate in PBS) and left in X-gal reaction substrate overnight at 37°C in the dark. Slides were next counterstained with nuclear fast red (VECTASHIELD®, Cat No. H-3403), dehydrated through an ethanol gradient, and embedded in mounting medium Xylene (Fisher Scientific).

### Alcian Blue Staining

Fixed samples were cryostat sectioned at 10 µm, washed in PBS to remove OCT, and incubated in alcian blue (0.03% alcian blue, 80% ethanol, 20% acetic acid) for 30 minutes. Stained sections were washed in running tap water for 2 minutes, then counterstained with nuclear fast red (VECTASHIELD®, Cat No. H-3403), dehydrated through an ethanol gradient and mounted in Mounting Medium Xylene (Fishier Scientific).

### Imaging

Fluorescent and bright-field images were taken using a ZEISS AxioPlan 2 microscope (Carl Zeiss MicroImaging LLC, NY, USA) with Axiovision 4.8. Images were subsequently refined in Adobe Photoshop.

### LCM and RNAseq

Tibial insertions of patellar tendons were obtained by laser capture micro-dissection (LCM) from 8 µm cryosections from two week-old knee joints. Knee joints were embedded in OCT, snap-frozen, and stored at −80°C. The embedded tissues were sectioned at −20°C at 8 µm, then mounted on membrane slides (Applied Biosystems®, Cat No.LCM0522), and stored at −80°C. Prior to laser capture microdissection, slides were fixed and processed through dehydration procedures using a HistoGene LCM frozen section staining kit (Applied Biosystems®, Cat No. KIT 0401). The tibial insertions of patellar tendon were cut by a Veritas microdissection instrument (model 704; Molecular Devices, Sunnyvale, CA) with the laser set at 2 µm, power 4.75. Sections were manually picked up using fine forceps under a dissection microscope. Total RNA from pooled sections from each animals (n = 3) was purified using a Arcturus® PicoPure® RNA isolation kit (Applied Biosystems®, Cat No KIT0204) following the manufacturer’s specification with DNase I treatment. RNA samples were then processed according to recommended procedures using the Ovation RNA-seq system V2 (Nugen, Cat No. 7102). Two control and 2 SmotKO RNAs were subjected to RNAseq procedures. Sequencing was performed using Illumina Hiseq2000 system according to Illumina protocols.

### RNA Isolation and Real-time Q-PCR

Four cultured PTTs were pooled and homogenized in sample lysis buffer, RLT buffer, from a RNeasy micro kit (Qiagen, Cat No. 74004) and total RNA was isolated following the manufacturer’s specifications (Qiagen, Cat No. 74004). cDNAs were synthesized using SuperScript™ III First-Strand synthesis System for Q-PCR (Invitrogen, Cat No. 18080-051). RNA isolation of section samples from LCM was described in the **LCM and RNAseq** sections.

cDNA samples were amplified with inventoried TaqMan® Gene Expression Assays (Table 3) and analyzed by a StepOnePlus Real-Time PCR system (Applied Biosystems). Reactions were analyzed in triplicate and expression levels were normalized to those of 18S ribosomal RNA (rRNA), which is stably expressed in tendons and joints [19], [38].

**Table 3:** TaqMan gene expression assays (Applied Biosystems) used for Q-PCR.

Gene	Assay ID
***18S***	Mm03928990_g1
***Aspn***	Mm00445945_m1
***Bgn***	Mm00455918_m1
***Col1a1***	Mm00801666_g1
***Col2a1***	Mm01309565_m1
***Gli1***	Mm00494645_m1
***Gpc3***	Mm00516722_m1
***Tnc***	Mm00495662_m1

### Biomechanical Testing

The mechanical properties of patella-patellar tendon-tibia samples were investigated in 12-week old female control (*Smof/+, Smof/−,or ScxCre;Smof/+*) (n = 12) and Smo loss-of-function mice (*ScxCre;Smo^f/−^* )(n = 5). Paired no. 11 surgical blades were gripped in a needle driver to dissect a consistent central width (0.59±0.02 mm, mean±SE) patella-PT-tibia complex from the right limb of each animal. Tendon complexes were failed in tension as previously described [39]. Briefly, tendon complexes were loaded in a custom grip, immersed in a 37°C phosphate buffered saline bath, and preloaded to 20 mN in a material testing system (100R; TestResouces; Shakopee, MN). Calibrated digital images were take in the sagittal and coronal planes to measure tendon thickness and width, respectively. Tendons were preconditioned (25 cycles, 0–1% strain, 0.003 mm/s) and failed in tension (0.003 mm/s) while recording grip-to-grip displacement and load. The failure mechanism (midsubstance v. insertion) was identified based upon the location of initial tissue tear observed in image sequences captured during testing. All data was collected by an observer blinded to the genotype of the specimen.

### Statistical Analysis

Differences between control and mutant or control and treatment groups were determined for biomechanical testing and real time-QPCR using t-test (*p*<0.05). Linear regression analysis was performed to evaluate correlations between animal body weight and each biomechanical or dimensional parameter, and logistic regression analysis was used to evaluate effects of genotype on tendon failure mechanism. Regression analyses were completed using SPSS 13.0 (Chicago, IL). Data from the semi-quantitative analysis were analyzed using one-way ANOVA followed by Student-Newman Keuls test (*p*<0.05)(Medcalc software). RNAseq data was analyzed using the Avadis NGS software. RNAseq data was normalized based on reads per kilobase exon per million mapped sequences (RPKM) and filtered based on expression, removing those that failed to have a minimum of 3 RPKM in at least one sample. The Audic Claverie test was used to find differentially expressed genes (*p*<0.05) with Benjamini Hochberg FDR multiple testing correction [40].

## Supporting Information

Figure S1 **Breeding scheme for conditional Smoothened (Smo) activation and inactivation. (A)** Mice expressing a constitutively active form of the Smo protein in tenocytes (CA-Smo) were generated by crossing ScxCre with a dominant active allele of Smo mouse (SmoM2). This dominate active allele is blocked by a loxP-flanked stop coden. Crossing ScxCre with SmoM2 mice will lead to constitutive activation of Hh signaling in tenocyte populations. **(B)** Smo tissue-specific knockout (*ScxCre;Smo^f/−^* or Smo tKO) mice can be generated by crossing *ScxCre;Smo^+/−^* with *Smo^f/f^*. After crossing these two mouse lines, tenocytes expressing the recombinase in their offspring that harbor heterozygous for the floxed and null alleles will delete the floxed allele of Smo but not in other cells. Thus, we can study the function of Smo-mediated signaling, Hh signaling, during tendon development.
(TIF)
