# Supplementary figures and images for: A Role for Hedgehog Signaling in the Differentiation of the Insertion Site of the Patellar Tendon in the Mouse
Source: PLoS One. 2013 Jun 10;8(6):e65411. doi: 10.1371/journal.pone.0065411 (PMC3677907; doi:10.1371/journal.pone.0065411)

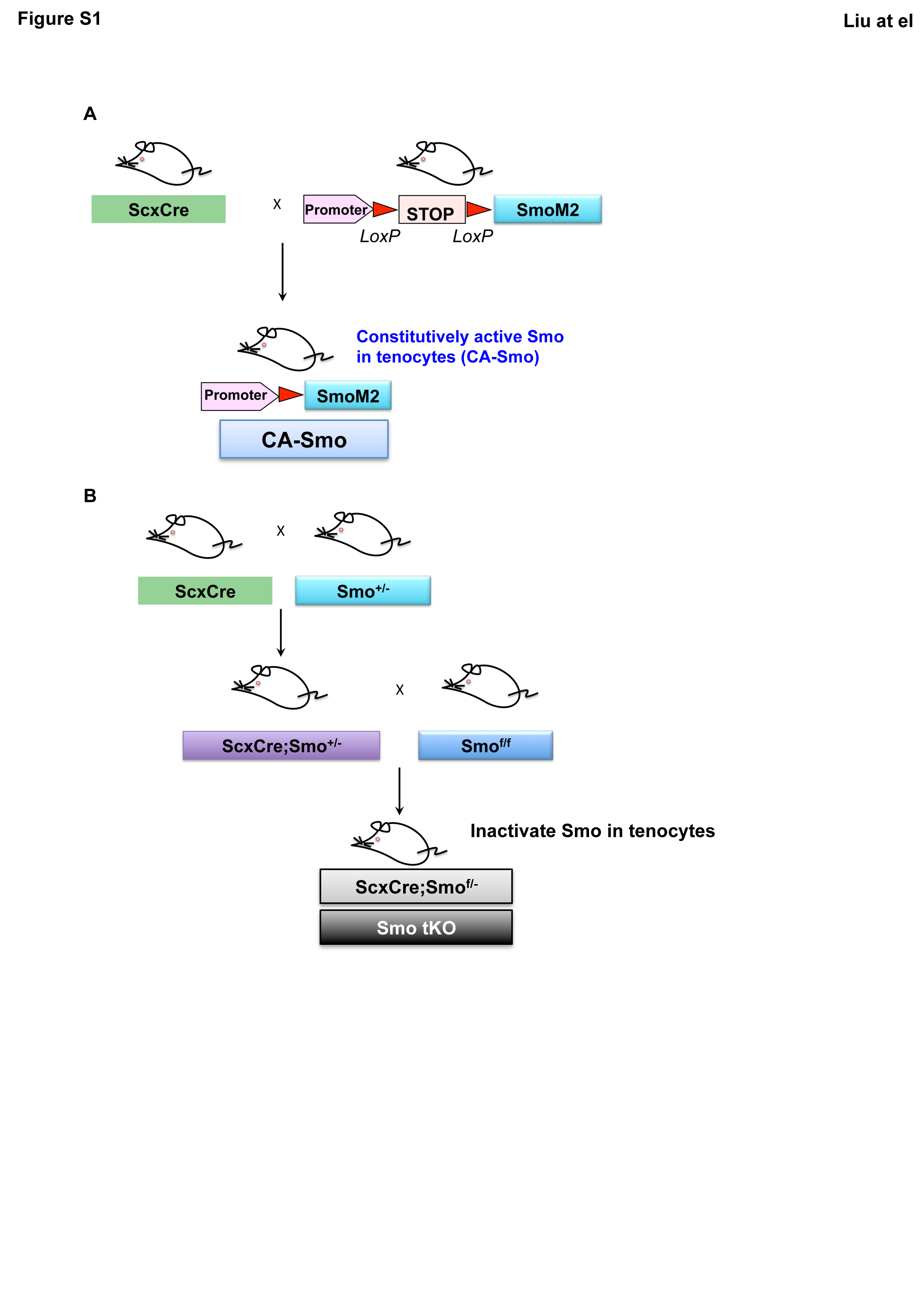

Supplement: Figure S1 — Breeding scheme for conditional Smoothened (Smo) activation and inactivation. (A) Mice expressing a constitutively active form of the Smo protein in tenocytes (CA-Smo) were generated by crossing ScxCre with a dominant active allele of Smo mouse (SmoM2). This dominate active allele is blocked by a loxP-flanked stop coden. Crossing ScxCre with SmoM2 mice will lead to constitutive activation of Hh signaling in tenocyte populations. (B) Smo tissue-specific knockout (ScxCre;Smof/− or Smo tKO) mice can be generated by crossing ScxCre;Smo+/− with Smof/f. After crossing these two mouse lines, tenocytes expressing the recombinase in their offspring that harbor heterozygous for the floxed and null alleles will delete the floxed allele of Smo but not in other cells. Thus, we can study the function of Smo-mediated signaling, Hh signaling, during tendon development. (TIF) [file pone.0065411.s001.tif]
